# Evaluation of the Hypothalamic–Pituitary–Adrenal Axis in Patients with Primary Sjögren’s Disease

**DOI:** 10.3390/medicina60111886

**Published:** 2024-11-17

**Authors:** Ana Glavina, Petar Zurak, Dinko Martinović, Majda Gotovac, Daniela Šupe-Domić, Liborija Lugović-Mihić

**Affiliations:** 1 Department of Dental Medicine, University Hospital of Split, 21000 Split, Croatia; glavina2014@gmail.com; 2 Department of Oral Medicine, Study of Dental Medicine, School of Medicine, University of Split, 21000 Split, Croatia; 3 Study of Dental Medicine, School of Medicine, University of Split, 21000 Split, Croatia; petarzurak1@gmail.com; 4 Department of Maxillofacial Surgery, University Hospital of Split, 21000 Split, Croatia; d.m.993@hotmail.com; 5 Department of Pathophysiology, School of Medicine, University of Split, 21000 Split, Croatia; 6 Teaching Public Health Institute of Split and Dalmatia Country, 21000 Split, Croatia; majda.gotovac@nzjz-split.hr; 7 Department of Medical Laboratory Diagnostics, University Hospital of Split, 21000 Split, Croatia; daniela.supedomic@gmail.com; 8 Department of Health Studies, University of Split, 21000 Split, Croatia; 9 Department of Dermatovenereology, University Hospital Center Sestre Milosrednice, 10000 Zagreb, Croatia; 10 School of Dental Medicine, University of Zagreb, 10000 Zagreb, Croatia

**Keywords:** biomarkers, cortisol, saliva, quality of life

## Abstract

*Background and Objectives:* Patients with primary Sjögren’s disease (pSjD) show contradictory results regarding the activity of the hypothalamic–pituitary–adrenal (HPA) axis. The aim of this study was to determine the salivary cortisol concentration to evaluate the function of the HPA axis (hypoactive/hyperactive) between patients with pSjD and control subjects. *Materials and Methods:* A total of 34 subjects participated in the cross-sectional study: 17 patients with pSjD and 17 control subjects. Stimulated whole saliva (SWS) was used to determine salivary cortisol concentration using an enzyme-linked immunosorbent assay (ELISA). *Results:* The salivary cortisol concentration showed a statistically significant difference between patients with pSjD and control subjects (4.69 ± 2.88 vs. 0.49 ± 0.37; *p* < 0.001; Student *t*-test). The area under the curve (AUC) was 100.0% in patients with pSjD (*p* < 0.001). The cut-off point was set to >1.454. The patients with pSjD had four times higher scores for depression and stress and six times higher scores for anxiety compared to the control subjects (*p* = 0.048, *p* < 0.001, *p* = 0.038; Mann–Whitney U test). The patients with pSjD had a statistically significantly higher total Oral Health Impact Profile (OHIP) score compared to the control subjects (*p* < 0.001, Mann–Whitney U test). *Conclusions:* The patients with pSjD showed short-term hyperactivity of the HPA axis compared to the control subjects.

## 1. Introduction

The systemic autoimmune disorder primary Sjögren’s disease (pSjD) is characterized by lymphocytic infiltration of the secretory glands, especially the lacrimal glands and salivary glands. This leads to *sicca* syndrome, which includes dryness of the eyes (*xerophthalmia*), oral cavity (*xerostomia*), pharynx, larynx (*xerotrachea*) and/or vagina. Patients with pSjD may also be characterized by extraglandular manifestations such as skin, musculoskeletal, pulmonary, renal, hematologic, and neurologic changes [1]. The spectrum of the disease ranges from an organ-specific autoimmune disease to a systemic disease. A systematic literature search in PubMed and Embase revealed that the combined incidence rate for pSjD is 6.92 per 100,000 people. The median age at diagnosis is 56.16 years [2]. The disease is ten times more common in middle-aged women than in men [3].

The disease usually has a mild clinical course. In a small number of patients, the disease takes a severe course with numerous systemic manifestations and a potentially poor outcome. A characteristic finding in the oral cavity is the presence of xerostomia as a result of hyposalivation. The consequences of xerostomia and hyposalivation are numerous: difficulty chewing, swallowing and speaking, altered taste, malnutrition, limited social interaction, difficulty wearing dentures, increased risk of caries (biting surfaces and tooth necks), oral candidiasis (usually chronic erythematous), and hyperlobulated tongue. Inspection may reveal painless swelling of the salivary glands, i.e., parotid glands and submandibular glands [4].

The differential diagnosis of Sjögren’s disease (SjD) covers a broad clinical spectrum of diseases: systemic lupus erythematosus (SLE), rheumatoid arthritis (RA), infections [hepatitis C virus (HCV), human immunodeficiency virus (HIV), cytomegalovirus (CMV)], various non-autoimmune causes of dry mouth or dry eyes (age, medications), IgG4 disease, sarcoidosis, amyloidosis, lymphoma, and graft-versus-host disease (GVHD). In view of the differential diagnosis, it is not advisable to make the diagnosis of pSjD solely on the basis of positive SSA/Ro and SSB/La autoantibodies. These autoantibodies can also be detected in other autoimmune diseases, but also in healthy people [5].

The hypothalamic–pituitary–adrenal (HPA) axis is one of the most important neuroendocrine systems responsible for mediating the stress response. Chronic stress leads to a dysfunction of the HPA axis. In all chronic stress-related diseases, the HPA axis is altered in such a way that it can no longer fulfill its immunoregulatory function, leading to the development of the disease. Although many studies show that cortisol levels are low under chronic stress conditions due to depletion of the HPA axis (in an effort to compensate for the response to a stressful stimulus), there is also evidence to the contrary [6].

The objective of this study was to determine the salivary cortisol concentration in patients with pSjD compared to control subjects, i.e., to assess the function of the HPA axis (hypoactive/hyperactive). The specific objectives of this study were to determine disease duration, levels of depression, anxiety and stress, quality of life (QoL), EULAR Sjögren’s Syndrome Disease Activity Index (ESSDAI) in patients with pSjD and their impact on salivary cortisol concentration. The hypothesis of this study was that a higher salivary cortisol concentration correlates with poorer mental health (higher levels of depression, anxiety and stress) and poorer QoL.

## 2. Materials and Methods

### 2.1. Study Design, Subjects, Inclusion and Exclusion Criteria

This cross-sectional study included a total of 34 subjects who were divided into two groups: 17 patients with pSjD and 17 control subjects. The control group consisted of randomly selected healthy patients (without oral and systemic diseases) from the Department of Diagnostic Radiology, Dental Clinic Split, Split, Croatia. This study was conducted from April to July 2024 at the Dental Polyclinic Split, the teaching base of the School of Medicine, University of Split (study of Dental Medicine), Split, Croatia. The study protocol was explained to each subject (verbally and in writing), and after signing the informed consent form, they were included in this study. The Ethics Committee of the School of Medicine, University of Split, Split, Croatia, approved the study protocol on 29 April 2024 (Class: 029-01/24-02/0001, Reg. no.: 2181-198-03-04-24-0044). This study was conducted in accordance with the principles of the Declaration of Helsinki (1964). All subjects who did not understand the nature and purpose of this study and those who did not agree with the content of the informed consent form were excluded from this study.

The inclusion criterion for patients with pSjD was:

1. Patients with pSjD diagnosed based on the American Colleague of Rheumatology (ACR) and European League Against Rheumatism (EULAR) diagnostic criteria [7].

The exclusion criteria for patients with pSjD were:

1. Patients suffering from systemic [diabetes, arterial hypertension, cardiovascular disease (CVD), kidney disease, liver disease, obesity] and/or other autoimmune diseases and/or cancer;
2. Pregnant women;
3. Patients who have received corticosteroid, immunosuppressive or psychoactive therapy (anxiolytics, anticonvulsants, antidepressants) in the last year;
4. Hormonal therapy;
5. Smoking;
6. Inflammatory diseases of the oral cavity (gingivitis, periodontitis).

### 2.2. Clinical and Laboratory Parameters

All subjects were clinically examined by the same oral medicine specialist (A.G.) with more than five years of experience. The medical history, medication and disease duration (in months) were recorded for all subjects. A sialometry test was performed to determine the amount of unstimulated whole saliva (UWS) and stimulated whole saliva (SWS) in patients with pSjD. The following parameters were recorded in patients with pSjD: focus score (FS) as a result of a minor labial salivary gland biopsy; presence of antinuclear antibodies (ANA); presence of anti-SSA/Ro60, anti-SSA/Ro52, anti-SSB/La; Schirmer test. A Schirmer test with a result of ≤ 5 mm/5 min in at least one eye was considered abnormal (positive). A UWS value ≤ 0.1 mL/min was compatible with the diagnosis of SjD.

### 2.3. Saliva Collection and Sampling

The SWS was used as a reference for the determination of salivary cortisol concentration in patients with pSjD and control subjects [8].

The SWS was collected according to the following protocol:

1. All subjects were asked not to expose themselves to intense physical or mental stress for three days prior to sampling;
2. All subjects were asked not to eat, drink, brush their teeth or smoke for 90 min prior to sampling;
3. SWS was collected in the morning hours (between 9 and 10 a.m.);
4. In women of reproductive age, SWS was collected during the follicular phase of the menstrual cycle [9];
5. Subjects were given a 1.0% solution of vitamin C (1 g ascorbic acid in 1 dcl water) to stimulate salivation [10];
6. Subjects collected 1.00 to 1.50 mL of saliva in graduated tubes (*Salivette*) (Ref. 51. 1534.500, SARSTEDT AG & Co. KG, Nümbrecht, Germany) using the “spit method” [11]. The subjects collected saliva in their mouths for 60 s and then spat it out into a graduated tube. The procedure was repeated for a further 10 min.

Saliva samples containing blood (by visual inspection) were excluded from this study [12,13].

The samples were delivered to the Department of Medical Laboratory Diagnostics, Clinical Hospital Center Split, Split, Croatia. They were first centrifuged at 1500× *g* for five minutes and then stored at −20 °C. To determine the salivary cortisol concentration, the frozen samples were first stored at room temperature for 30 min. They were then centrifuged again at 1500× *g* for five minutes. The salivary cortisol concentration was analyzed using the immunochemical ELISA method with reagents from EUROIMMUN (Medizinische Labordiagnostika AG, Lübeck, Germany). The lower sensitivity limit of the salivary cortisol concentration tested by the manufacturer is 0.15 ng/mL; the linearity ranges from 0.10 to 28.30 ng/mL. The coefficient of variation in the series was 3.70, 4.20, and 3.20 for the concentrations 0.60, 2.10, and 13.40 ng/mL, and between the series 9.70, 7.90, and 4.70 for the concentrations 1.30, 2.80, and 13.90 ng/mL. The saliva samples were analyzed with the Elysis Duo device (Human, Wiesbaden, Germany).

### 2.4. Instruments

#### 2.4.1. Depression, Anxiety and Stress Scale (DASS-21)

Mental health was assessed using the Croatian validated version of the DASS-21, the Croatian adaptation of Jokić-Begić, Jakšić, Ivezić and Surányi (2012). The same researcher (A.G.) asked each subject 21 statements to ensure that they understood them correctly. The statements reflected the level of depression, anxiety and stress in the last seven days. Each scale (depression, anxiety, stress) comprised seven statements. Respondents answered on a Likert scale ranging from 0—the statement is not true to 3—the statement is completely or mostly true. The total result is calculated by adding up the points for all statements in the depression, anxiety and stress subscales and multiplying the resulting number by two. Higher total scores reflect poorer mental health. The DASS-21 is a psychometrically high-quality instrument, which is why it is widely used in clinical practice and research [14].

#### 2.4.2. Croatian Oral Health Impact Profile Questionnaire (OHIP-CRO14)

The OHIP-14, i.e., its Croatian version, was used to assess the impact of oral health on QoL. The OHIP-14 is a general measure of QoL related to oral health and not a specific measure of the condition/disease. The same researcher (A.G.) asked all respondents 14 questions about QoL a month ago. The 14 questions can be divided into seven groups: functional limitation, physical pain, psychological discomfort, physical impossibility, psychological impossibility, social impossibility, and handicap. The seven groups can be further subdivided into three categories: physical factor (functional limitation, physical pain and impossibility), psychological factor (psychological discomfort and impossibility) and social factor (social impossibility and handicap). The respondents answered the statements on a Likert scale from 0—never to 4—very often. The result of the questionnaire is determined by simply adding up the answers, with higher results indicating poorer oral health, i.e., a poorer QoL [15].

#### 2.4.3. The ESSDAI

The ESSDAI is a systematic objective index of disease activity in patients with pSjD, a heterogeneous disease that can affect multiple organ systems. The ESSDAI is considered the “gold standard” for the assessment of disease activity. The ESSDAI comprises 12 domains, i.e., organ systems: musculature, hematology, kidneys, glands, skin, peripheral nervous system (PNS), central nervous system (CNS), respiratory system, joints, constitution, lymphadenopathy and biology. Each system is divided into 3 or 4 levels of activity. The total ESSDAI is calculated from the sum of all systems (maximum 32). The activity levels for each system range from 0—no activity to 4—high activity [16].

### 2.5. Statistical Analysis

The sample size analysis was performed using data from a pilot study with 8 pSjD patients and 8 matched control subjects. The salivary cortisol concentration, which was the main outcome of this study, was used for the calculation. The mean salivary cortisol concentration was 3.54 ± 2.78 compared to 0.53 ± 0.28 ng/mL in the control group. With a type I error of 0.05 and a power of 90.0%, the required sample size was 11 subjects per group.

The analysis of the collected data was performed with the statistical program MedCalc (MedCalc Software, Ostend, Belgium, version 22.030). All continuous quantitative variables are presented as mean ± standard deviation (SD), while non-continuous variables are presented as median (interquartile range, IQR). All qualitative variables are presented as whole numbers and percentages. The normality of the distribution was tested using the Kolmogorov–Smirnov test. The comparison of the continuous variables between the groups was carried out using the Student t-test, while the non-continuous variables were compared using the Mann–Whitney U test. The categorical variables were compared between the groups using the chi-square test. The correlation was calculated for continuous variables with Pearson’s correlation and for non-continuous variables with the Spearman correlation. The diagnostic performance of salivary cortisol in determining whether a subject had SjD was assessed using the area under the receiver operating curve (AUROC). The level of statistical significance was set at *p* < 0.05.

## 3. Results

### 3.1. Clinical and Laboratory Parameters

The mean age of the patients with pSjD (N = 17) was 56.4 ± 16.8 years and 57.0 ± 16.0 years in the control group. Both groups consisted of female subjects (94.1%). There was no statistically significant difference in gender and age between patients with pSjD and control subjects (*p* = 0.898, *p* = 0.925). The mean disease duration in patients with pSjD was 23.1 ± 17.3 months.

The patients with pSjD had a statistically significantly higher salivary cortisol concentration compared to the control subjects (*p* < 0.001). The patients with pSjD had a five times higher salivary cortisol concentration than the control subjects (Table 1).

### 3.2. Psychological Profile

The patients with pSjD had statistically significantly higher scores for depression, anxiety and stress compared to the control subjects (*p* = 0.048, *p* < 0.001, *p* = 0.038). The patients with pSjD had four times higher scores for depression and stress and six times higher scores for anxiety than the control subjects. Stress was the most important mental disorder in patients with pSjD (Table 2).

### 3.3. QoL

The patients with pSjD had a statistically significantly higher OHIP-CRO14 total score (poorer QoL) compared to the control subjects (*p* < 0.001). The patients with pSjD had statistically significantly higher scores in the OHIP-CRO14 dimensions of functional limitation, physical pain, physical impossibility, psychological impossibility and handicap compared to the control subjects (Table 3).

### 3.4. Receiver Operating Curve (ROC) for Salivary Cortisol

**Figure 1:**
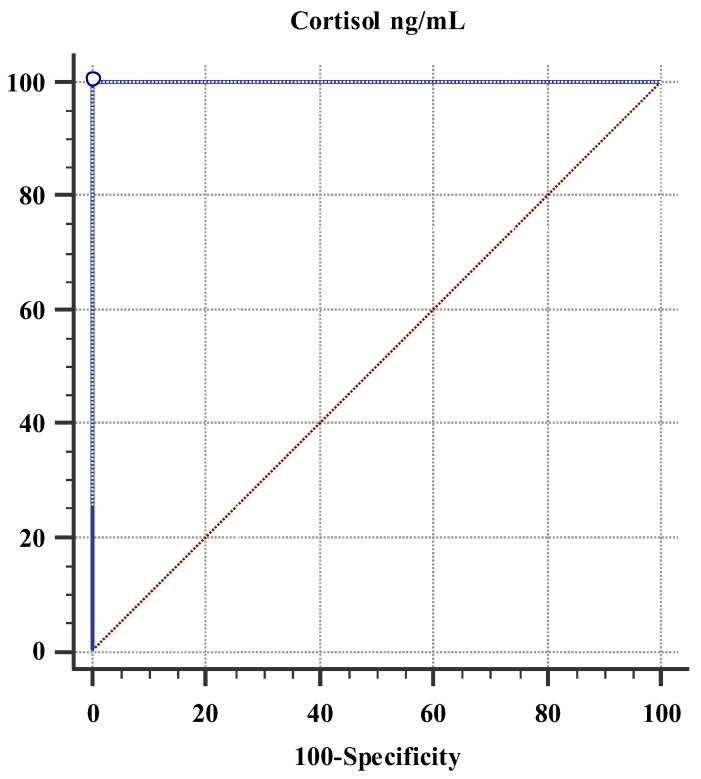
ROC for salivary cortisol concentration in patients with pSjD. Data: AUC: 1.00 (0.897–1.000). *p* < 0.001. Youden cut-off: >1.454 ng/mL. Sensitivity: 100.0%. Specificity: 100.0% Abbreviations: AUC, area under the curve; pSjD, primary Sjögren’s disease; ROC, receiver operating curve.

The ROC was used to determine the cut-off point for the diagnostic value of salivary cortisol concentration in patients with pSjD. The area under the curve (AUC) was 100.0% (*p* < 0.001) in patients with pSjD. This was acceptable for distinguishing patients with pSjD from control subjects. The cut-off point was set at >1.454 for patients with pSjD. The sensitivity was 100.0% and the specificity was 100.0% (Figure 1).

### 3.5. Salivary Cortisol

There was no statistically significant correlation between salivary cortisol and clinical parameters (UWS, SWS, ESSDAI, disease duration) in patients with pSjD (Table 4).

There was no statistically significant correlation between salivary cortisol and the psychological profile and QoL in patients with pSjD. However, there was a good positive statistically significant correlation between salivary cortisol and stress in the control group (r = 0.621, *p* = 0.007) (Table 5).

### 3.6. The ESSDAI

There was a good negative statistically significant correlation between the ESSDAI and the OHIP-CRO14 dimension of psychological discomfort (r = −0.474, *p* = 0.049) (Table 6).

### 3.7. Disease Duration

There was a good negative statistically significant correlation between disease duration and the OHIP-CRO14 dimension of psychological discomfort in patients with pSjD (r = −0.690, *p* = 0.002) (Table 7).

## 4. Discussion

The patients with pSjD showed short-term hyperactivity of the HPA axis as measured by the concentration of the salivary stress biomarker (cortisol). We also investigated the influence of different parameters (UWS, SWS, ESSDAI, disease duration, psychological profile, QoL) on salivary cortisol concentration in patients with pSjD. The hypothesis of the present study was not confirmed by the results, i.e., a statistically significant correlation of salivary cortisol with the psychological profile and QoL in patients with pSjD could not be demonstrated.

The patients with pSjD had a five times higher salivary cortisol concentration compared to the control subjects (*p* < 0.001). There are few studies on salivary cortisol concentrations in patients with pSjD (and other autoimmune diseases), and the results are contradictory. The study by Montero-López E et al. is consistent with the present study. Their study involved 35 women with various autoimmune diseases [SjD, SLE, systemic sclerosis (SSc)] and 30 healthy subjects. They monitored the salivary cortisol concentrations during the day (five time points) and the hair cortisol concentrations over three months to avoid daily fluctuations and influences on the results. The patients with autoimmune diseases (SjD, SLE, SSc) had a statistically significantly higher daily salivary cortisol concentration and hair cortisol over a period of three months than the control subjects [17].

The study by Jung J-Y et al. showed no statistically significant difference in salivary cortisol concentrations between patients with autoimmune diseases (N = 100) and control subjects (N = 49) [18]. The study by Miller BE et al. also showed no statistically significant difference in salivary cortisol concentrations between patients with SjD and control subjects [19]. The discrepancies in the results can be explained by the heterogeneity of the groups, different times of salivary cortisol collection, different wake-up times of the subjects and different sample sizes.

The patients with pSjD had statistically significantly poorer mental health (higher scores for depression, anxiety and stress) compared to the control subjects (*p* = 0.048, *p* < 0.001, *p* = 0.038). Stress was the most important mental disorder in patients with pSjD. The results of the present study are partially consistent with the findings of Milic V et al. They showed that patients with pSjD had statistically significantly higher anxiety scores (but no depression) compared to control subjects [20]. Cui Y et al. conducted a study with 160 patients with pSjD and 170 control subjects. The patients with pSjD had statistically significantly higher scores for depression (36.9%) and anxiety (33.8%) compared to the control subjects [21]. Considering the multiple pathology of the disease with many systemic manifestations, the above studies are likely to support the findings suggesting a disturbed psychological profile in patients with pSjD.

The patients with pSjD had a statistically significantly poorer QoL (higher OHIP-CRO14 scores) compared to the control subjects (*p* < 0.001). The physical factor category was affected in all three dimensions of the OHIP-CRO14 (functional limitation, physical pain and impossibility). This shows that xerostomia and hyposalivation have a significant negative impact on QoL in patients with pSjD. Oral medicine specialists need to be part of the multidisciplinary team of pSjD patients [22]. The results of other studies are consistent with those of the present study. Serrano J et al. showed that xerostomia and reduced UWS levels statistically significantly reduced QoL in 61 patients with pSjD [23]. Glavina A et al., Azuma N et al., Gobeljić MŠ et al., Rusthen S et al. and a systematic review by Schmalz G et al. showed a statistically significant poorer QoL in patients with SjD compared to control subjects [22,24,25,26,27]. This clearly confirms the negative impact of pSjD on patients’ QoL.

There was no statistically significant correlation between salivary cortisol and clinical parameters (UWS, SWS, ESSDAI, disease duration) in patients with pSjD. Miller BE et al. also showed no statistically significant correlation between salivary cortisol and whole saliva volume [19]. In the present study, there was no statistically significant correlation between the ESSDAI and the QoL (OHIP-CRO14 total score) in patients with pSjD. There was a good negative statistically significant correlation between the ESSDAI and the OHIP-CRO14 dimension of psychological discomfort (r = −0.474, *p* = 0.049). In contrast to the results of the present study, Dias LH et al. found no statistically significant correlation between the ESSDAI and QoL in 77 patients with pSjD [28]. This can be explained by the fact that the ESSDAI assesses the current disease activity (and not the organic dysfunction that has already occurred). Therefore, the patient’s perception of QoL was not changed at the observed time point.

There was a good negative statistically significant correlation between disease duration and the OHIP-CRO14 dimension of psychological discomfort in patients with pSjD (r = −0.690, *p* = 0.002). There was no statistically significant correlation between disease duration and psychological profile in patients with pSjD. This indicates good adaptive coping mechanisms in dealing with the autoimmune disease and its consequences in patients with pSjD. The results of the present study are not consistent with the findings of Zhang Y et al. They showed that longer disease duration leads to more intense disease symptoms, i.e., more physical incapacity and fatigue [29]. The discrepancies in the results may be due to a small sample size and patients with different degrees of disease activity (as well as the use of different instruments/indexes in the assessment of mental health, QoL and disease activity).

The advantage of the present study is that one of the exclusion criteria was drug therapy (corticosteroids, immunosuppressants, psychotropic drugs) one year ago. It is known that a shorter period of time can influence the concentration of salivary biomarkers [30].

The present study has several limitations. This is a cross-sectional study (single-centre) with a small number of subjects (pilot study), which makes it impossible to draw causal conclusions. The patients with pSjD were treated as outpatients and had lower systemic involvement (lower ESSDAI values). The salivary cortisol concentration was measured at a single time point (no influence on the subjects’ wake-up time possible). In the control group, there was a good positive statistically significant correlation between salivary cortisol and stress (r = 0.621, *p*= 0.007). Such a result in the control group (stressor) probably influenced the results of the present study, which must be interpreted in this context. It is necessary to include a larger number of patients and subdivide them on the basis of extraglandular manifestations. Future studies should also include other stress biomarkers (such as alpha-amylase) that have an impact on the HPA axis and mediate the body’s response to stress. Further prospective longitudinal studies with strictly defined methodology are needed.

## 5. Conclusions

The patients with pSjD showed a five times higher salivary cortisol concentration, i.e., a short-term hyperactivity of the HPA axis compared to the control subjects. The patients with pSjD had poorer mental health (four times higher scores for depression and stress and six times higher scores for anxiety) compared to the control subjects. The patients with pSjD had a poorer QoL, with the category of physical factor (functional limitation, physical pain and impossibility) being particularly affected compared to the control subjects. The determination of salivary cortisol can serve as an additional diagnostic method (rapid and non-invasive) and indicate the need for an additional therapeutic approach (psychological, psychiatric) in patients with pSjD.

## Figures and Tables

**Table 1:** Demographic, clinical and laboratory data in patients with pSjD and control subjects.

Parameter	pSjD Group (N = 17)	Control Group (N = 17)	*p*
Female (N, %)	16 (94.1)	16 (94.1)	0.898 *
Age (years)	56.4 ± 16.8	57.0 ± 16.0	0.925 ^†^
Disease duration (months)	23.1 ± 17.3	-	-
FS	1.07 ± 0.82		
ESSDAI	1.0 (0.0–2.0)	-	-
Schrimer (N, %)	10 (58.8)	-	-
UWS (ml/min)	0.06 ± 0.11	-	-
SWS (ml/min)	0.20 ± 0.22	-	-
Dry eyes (N, %)	12 (70.6)	-	-
Dry mouth (N, %)	15 (88.2)	-	-
ANA (N, %)	13 (76.5)	-	-
SSA/Ro52 (N, %)	10 (58.8)	-	-
SSA/Ro60 (N, %)	9 (52.9)	-	-
SSB (N, %)	6 (35.3)	-	-
Cortisol (ng/mL)	4.69 ± 2.88	0.49 ± 0.37	<0.001 ^†^

All data are given as whole numbers (percentage), arithmetic mean ± standard deviation or median (interquartile range). * Chi-square test. ^†^ Student *t*-test. Mann–Whitney U test. Abbreviations: ANA, antinuclear antibodies; ESSDAI, EULAR Sjögren’s Syndrome Disease Activity Index; FS, focus score; pSjD, primary Sjögren’s disease; SAA/Ro52, SSA/Ro60, anti-Sjögren’s-syndrome-related antigen A autoantibodies; SSB, anti-Sjögren’s-syndrome-related antigen B autoantibodies; SWS, stimulated whole saliva; UWS, unstimulated whole saliva.

**Table 2:** Comparison of the psychological profile between patients with pSjD and control subjects.

Parameter	pSjD Group (N = 17)	Control Group (N = 17)	*p* *
Depression	4.0 (0.0–14.0)	1.0 (0.0–2.5)	0.048
Anxiety	6.0 (4.0–10.5)	1.0 (0.0–4.0)1	<0.001
Stress	12.0 (2.0–20.5)	3.0 (1.0–8.0)	0.038

All data are given as the median (interquartile range). * Mann–Whitney U test. Abbreviation: pSjD, primary Sjögren’s disease.

**Table 3:** Comparison of QoL between pSjD patients and control subjects.

Parameter	pSjD Group (N = 17)	Control Group (N = 17)	*p* *
OHIP-CRO14	28.0 (4.0–21.25)	0.0 (0.0–3.5)	<0.001
Functional limitation	2.0 (0.75–4.0)	0.0 (0.0–0.0)	<0.001
Physical pain	1.0 (0.0–4.0)	0.0 (0.0–0.0)	0.003
Psychological discomfort	2.0 (0.75–3.25)	0.0 (0.0–2.25)	0.067
Physical impossibility	1.0 (0.0–3.25)	0.0 (0.0–0.0)	0.004
Psychological impossibility	1.0 (0.0–1.25)	0.0 (0.0–0.0)	0.032
Social impossibility	0.0 (0.0–0.0)	0.0 (0.0–0.0)	0.116
Handicap	0.0 (0.0–2.25)	0.0 (0.0–0.0)	0.005

All data are given as the median (interquartile range). * Mann–Whitney U test. Abbreviations: OHIP-CRO14, Croatian version of the Oral Health Impact Profile-14 questionnaire; pSjD, primary Sjögren’s disease; QoL, Quality of Life.

**Table 4:** Correlation of salivary cortisol with clinical parameters in patients with pSjD.

Parameter	pSjD Group (N = 17)	
	r	*p*
UWS	−0.217 ^†^	0.402
SWS	−0.294 ^†^	0.252
ESSDAI	0.209 *	0.421
Disease duration (months)	−0.070 ^†^	0.787

* Spearman’s correlation coefficient. ^†^ Pearson’s correlation coefficient. Abbreviations: ESSDAI, EULAR Sjögren’s Syndrome Disease Activity Index; pSjD, primary Sjögren’s disease; SWS, stimulated whole saliva; UWS, unstimulated whole saliva.

**Table 5:** Correlation of salivary cortisol with QoL and psychological profile in patients with pSjD and control subjects.

Parameter	pSjD Group (N = 17)		Control Group (N = 17)	
	r *	*p*	r *	*p*
OHIP-CRO14	0.280	0.275	−0.066	0.800
Functional limitation	−0.158	0.544	−0.192	0.460
Physical pain	0.289	0.259	0.065	0.805
Psychological discomfort	0.177	0.497	0.134	0.609
Physical impossibility	0.231	0.372	0.051	0.845
Psychological impossibility	0.388	0.123	−0.142	0.586
Social impossibility	−0.039	0.882	0.001	0.989
Handicap	0.218	0.399	0.001	0.989
Depression	0.181	0.487	0.221	0.392
Anxiety	0.311	0.223	0.187	0.472
Stress	0.295	0.250	0.621	0.007

* Spearman’s correlation coefficient. Abbreviations: OHIP-CRO14, Croatian version of the Oral Health Impact Profile-14 questionnaire; pSjD, primary Sjögren’s disease; QoL, Quality of Life.

**Table 6:** Correlation of the ESSDAI with QoL and psychological profile in patients with pSjD.

Parameter	pSjD Group (N = 17)	
	r *	*p*
OHIP-CRO14	0.194	0.456
Functional limitation	0.228	0.379
Physical pain	0.450	0.069
Psychological discomfort	−0.474	0.049
Physical impossibility	0.461	0.062
Psychological impossibility	0.197	0.448
Social impossibility	0.328	0.199
Handicap	0.014	0.958
Depression	−0.367	0.147
Anxiety	−0.109	0.678
Stress	−0.143	0.582

* Spearman’s correlation coefficient. Abbreviations: ESSDAI, EULAR Sjögren’s Syndrome Disease Activity Index; OHIP-CRO14, Croatian version of the Oral Health Impact Profile-14 questionnaire; pSjD, primary Sjögren’s disease.

**Table 7:** Correlation of disease duration with QoL and psychological profile in patients with pSjD.

Parameter	pSjD Group (N = 17)	
	r *	*p*
OHIP-CRO14	0.014	0.958
Functional limitation	0.016	0.950
Physical pain	0.360	0.156
Psychological discomfort	−0.690	0.002
Physical impossibility	0.391	0.120
Psychological impossibility	0.098	0.708
Social impossibility	0.044	0.865
Handicap	−0.061	0.815
Depression	−0.014	0.956
Anxiety	0.144	0.581
Stress	0.065	0.803

* Spearman’s correlation coefficient. Abbreviations: OHIP-CRO14, Croatian version of the Oral Health Impact Profile-14 questionnaire; pSjD, primary Sjögren’s disease; QoL, Quality of Life.

## Data Availability

The data analyzed in the current study are available upon reasonable request by email to the corresponding author.
